# Delta Neutrophil Index as a New Marker of Purulent Inflammation in Men With Non-odontogenic Abscesses of the Neck

**DOI:** 10.7759/cureus.47165

**Published:** 2023-10-16

**Authors:** Yanko G Yankov

**Affiliations:** 1 Maxillofacial Surgery, University Hospital St. Marina, Varna, BGR; 2 General and Operative Surgery, Medical University “Prof. Dr. Paraskev Stoyanov”, Varna, BGR

**Keywords:** purulent inflammation, inflammation marker, maxillofacial surgery, oral surgery, neck, phlegmon, abscess, non-odontogenic abscess, inflammation, delta neutrophil index

## Abstract

Introduction

If non-odontogenic abscesses and phlegmons (all purulent inflammations where the etiology is not a diseased tooth) of the neck are not promptly treated, they can lead to serious complications and even end in the death of the affected patient. Classical markers of inflammation such as plasma concentration of leukocytes (WBC), neutrophils (Neu), C-reactive protein (CRP), and erythrocyte sedimentation rate (ESR) are elevated in inflammatory neck diseases, but none of them has been proven as a definite marker in the prediction of this type of pathology. This necessitates the search and analysis of new indicators that could be used in the diagnosis, follow-up, and prognosis of patients with purulent neck infections. Potentially, such a marker could be the delta neutrophil index (DNI), which is increasingly entering clinical practice as a prognostic indicator in critically ill patients with life-threatening illnesses, including sepsis and systemic inflammatory response syndrome (SIRS). In the world literature, there are no data that have been studied in patients with purulent diseases of the neck of non-odontogenic origin, which is the aim of this original article.

Materials and methods

This retrospective study included 40 men with an average age of 46 (18-87) years with non-odontogenic abscesses and phlegmons of the neck who were hospitalized and operated on. In all of them, the concentration of leukocytes, eosinophils (Eo), neutrophils, and mature polymorphonuclear neutrophil leukocytes (PMN) was examined on an automatic 5-Diff hematology analyzer, ADVIA 2120i (Siemens Medical Solutions USA, Malvern, PA). Thus, it was calculated according to the formula for calculating DNI (DNI% = (Neu%+Eo%) - PMN%). Retrospectively, 30 healthy men with an average age of 42 (18-81) years were used as a control group, in which the same indicators were examined during a preventive examination.

Results and discussion

Comparing the mean values of WBC, Neu, and DNI between the studied patients with non-odontogenic purulent neck infections (n=40) and the healthy male controls (n=30) it was found that all three indicators of inflammation are significantly higher in the ill men, and these differences are statistically significant (p<0.05): 10.19 ±2.68x10^3^/L versus 7.37 ±1.93x10^3^/L for leukocytes, 7.68 ±2, 76x10^3^/L versus 4.13 ±1.48x10^3^/L for neutrophils, and 1.11±0.83% versus -1.07±1.22% for DNI. Therefore, the high mean numbers of measured WBC and Neu in men with non-odontogenic purulent neck infections were associated with an increase in their mean calculated DNI. This gives us reason to think that while WBC and Neu alone are not sufficient for definitive diagnosis, treatment follow-up, and prediction of disease outcome, in combination with DNI they become reliable indicators in purulent neck infections.

Conclusions

The DNI correlates well with other well-known and established indicators of inflammation, such as the concentration of leukocytes and neutrophils in the peripheral blood of patients. Its calculation is fast as an implementation procedure and is economically beneficial. Its independent use in the diagnosis and treatment of these diseases is about to be investigated and analyzed.

## Introduction

According to data from our medical practice as maxillofacial and oral surgeons, the most common emergency conditions in the maxillofacial region, which end in hospitalization and surgical treatment of patients, are purulent inflammations of the soft tissues of the head and neck, called abscesses, and their wide-based spreads, called phlegmons. If they are not promptly treated, especially phlegmons, they can lead to serious complications and end in death [1]. The clinical picture in these diseases can be very diverse, from local signs of inflammation and slight discomfort in milder forms to difficulty in the normal course of vital functions, such as acute respiratory distress syndrome (ARDS) from compression of the upper respiratory tract by the collected purulent exudate, to intoxication, sepsis, and coma in severe cases [2,3]. Classical markers of inflammation such as plasma concentration of leukocytes (WBC), neutrophils (Neu), and C-reactive protein (CRP) are elevated in inflammatory neck diseases, but none of them has been proven as a definitive marker for prognosis in this type of pathology [4]. This necessitates the search and analysis of new indicators that could be used in the diagnosis, follow-up, and prognosis of patients with purulent neck infections. The delta neutrophil index (DNI) is increasingly entering medical practice as a prognostic indicator in critically ill patients with life-threatening diseases such as sepsis and systemic inflammatory response syndrome (SIRS) [5,6], acute abdomen [7], acute pyelonephritis [8], acute graft rejection in kidney transplant patients [9,10], acute gout attack [11], and septic arthritis [11,12]. In the world literature, there are no data that have been studied on infectious diseases of the neck of non-odontogenic origin, which is the purpose of this original article.

## Materials and methods

The present study is retrospective. For a period of nine months (from the beginning of July 2021 to the end of March 2022) in the Clinic of Maxillofacial Surgery at the University General Hospital for Active Treatment "St. Marina", Varna, Bulgaria, 41 men with non-odontogenic purulent inflammations in the neck area were hospitalized and operated on. The criteria for inclusion in the study were that the men had no anamnestic or physical evidence of the presence of another infectious disease (including mycotic and parasitic), except for the abscess or phlegmon of the neck for which they were hospitalized, the presence of imaging evidence of a purulent infection of the neck, and, during the operative intervention, the presence of purulent exudate in the neck area to be proven to be over 18 years of age. In all of them, the diagnosis of abscess or phlegmon in the neck was confirmed both by performing an imaging study (ultrasound, computed tomography with or without contrast, or nuclear magnetic tomography of the neck), in which a pus collection was visualized. This was the reason for surgical treatment, during which a different amount of purulent exudate was evacuated from all of them. Exclusion criteria from the study are all diseases and conditions that would lead to elevated values of the examined blood cells: recent surgical interventions, multiple traumas, cardiogenic shock, burns, pyrexia, the presence of evidence of infections (including mycotic and parasitic), oncological diseases, paraneoplastic syndrome, diseases leading to hypoperfusion (pneumonia, bronchial asthma), and the intake of medications, the intake of which leads to the release of cytokines like granulocyte colony-stimulating factor (G-CSF). Thus, one patient was excluded from the study due to the presence of an upper respiratory tract infection. The remaining 40 men had an average age of 46 years, ranging between 18 and 87 years.

In all of them, as a mandatory pre-operative examination, venipuncture from the median cubital vein in the cubital fossa is done, and blood is taken after preliminary disinfection of the skin above the venipuncture site. Hematology vacutainers are used, which are identified by a purple cap and have dipotassium ethylene diamine tetraacetic acid (K2 EDTA) as an additive sprayed on the inside of the vacutainer walls or tripotassium ethylene diamine tetraacetic acid (K3 EDTA) as a liquid in them. which do not allow the blood to clot, an important condition for the hematological analyst's ability to determine the concentrations of the blood cells being examined. The volume of the vacutainers themselves (2, 3, 6, or 7 ml) does not matter. It would be a mistake to use another type of vacutainer that would allow the blood to clot. Blood in our study was examined on an automatic 5-Diff hematology analyzer, ADVIA 2120i (Siemens Medical Solutions USA, Malvern, PA), in the central clinical laboratory of the same medical institution. Thus, in all patients, the numerical percentage values (%) of the plasma concentration of eosinophils (Eo), Neu, and mature polymorphonuclear neutrophil leukocytes (PMN) were obtained, from which the DNI was then calculated according to the formula for this: DNI% = (Neu%+Eo%) - PMN% [5,6,11]. Leukocyte and neutrophil counts were measured as counts per liter (x10^3^/L) and DNI as percentage (%).

Retrospectively, 30 healthy men with a mean age of 42 years, ranging between 19 and 81 years, from whom venous blood was collected and processed in the same way during preventive examinations in the fall of 2021, were used as a control group. The criteria for inclusion in the control group are 18 years of age and the absence of any acute diseases, including infectious ones. Exclusion criteria are age below 18 years, the presence of any diseases at the time of the examination, and 30 days before.

Data from this original article presented in numerical values were statistically processed with IBM SPSS software for Windows 7.0 (IBM Corp., Armonk, NY) and presented as their mean value ± standard deviation (SD). Their analysis was performed with the Student's t-test. Those values with p<0.05 were considered significant.

## Results

The results of the examination of the venous blood of the studied patients are presented in Table 1.

**Table 1:** Values of the studied WBC, Neu, and DNI in patients with non-odontogenic abscesses WBC: leukocytes; Neu: neutrophils; DNI: delta neutrophil index

Number by order	Age (years)	WBC (х10^3^/L)	Neu (х10^3^/L)	DNI (%)
1	18	12.15	9.89	1.6
2	18	8.42	6.31	0.7
3	19	8.84	5.53	1.1
4	21	7.12	5.04	0.6
5	25	12.01	10.27	1.1
6	26	13.02	11.06	1.1
7	27	9.98	6.98	1.2
8	27	8.51	4.03	1
9	29	12.87	11.35	1.3
10	31	13.32	8.82	0.5
11	32	10.05	7.98	1.1
12	32	6.89	4.19	1.6
23	33	13.23	11.82	1.1
14	34	13.01	11.56	0.9
15	35	8.27	6.21	1
16	41	9.21	7.13	0.9
17	45	9.73	6.92	0.5
18	45	7.69	5.99	1.5
19	47	7.69	5.12	1.4
20	47	9.25	6.89	1.1
21	47	7.07	4.99	1.2
22	48	7.55	4.18	1.2
23	48	8.36	5.42	1.4
24	48	9.26	6.33	2.6
25	49	7.02	5.02	2.3
26	50	8.26	5.6	1
27	51	5.98	3.06	0.7
28	53	7.77	5.84	0.6
29	55	13.01	10.56	2.1
30	55	14.22	9.01	2
31	56	6.99	5	1.1
32	56	11.4	8.35	0.4
33	64	12.16	8.65	1.7
34	66	8.23	6.05	0.7
35	66	10.71	8.39	-1.5
36	68	16.46	13.41	1.6
37	70	14.09	11.95	-1.1
38	84	15.71	14.1	2.9
39	86	12.05	10.02	-0.7
40	87	10.08	7.99	0.8

Table 2 shows the values of the same indicators in men from the control group.

**Table 2:** Values of the studied WBC, Neu, and DNI in the healthy men from the control group WBC: leukocytes; Neu: neutrophils; DNI: delta neutrophil index

Number by order	Age (years)	WBC (х10^3^/L)	Neu (х10^3^/L)	DNI (%)
1	19	8.2	4.2	-0.71
2	24	7.72	3.86	-1.9
3	26	4.87	2.49	-0.1
4	26	6.1	3.62	-1.8
5	27	8.28	4.74	-3.2
6	27	9.36	4.73	-1.4
7	29	10.23	6.07	0.1
8	31	5.62	3.2	-1.3
9	32	12.51	8.21	-0.1
10	32	5.07	2.93	0.5
11	35	5.13	2.8	-1.1
12	37	8.12	4.4	0.1
13	37	9.41	4.68	-0.2
14	38	7.4	3.53	-0.2
15	40	6.43	2.84	-3.2
16	41	7.44	4.04	1
17	42	10.54	6.81	-1.4
18	44	4.9	2.9	-0.9
19	45	10.07	7.92	-1.4
20	46	7.5	3.5	-0.5
21	47	8.45	4.34	-0.7
22	79	8.52	4.57	-2.5
23	51	6.46	3.04	-2.9
24	53	7.4	4.2	-2.8
25	53	6.71	3.8	-1.6
26	55	6.4	3.9	0.4
27	65	6.16	4.54	0.4
28	65	6.38	3.04	-2.5
29	67	4.04	1.72	-2.7
30	81	5.56	3.2	0.56

Reference values for men aged 18 years and over are as follows: for WBC, from 13.23x10^3^/L to 10.33x10^3^/L; for Neu, from 11.82x10^3^/L to 7.00x10^3^/L. The DNI in healthy individuals is 0.0%.

The DNI is the subtraction between the fraction of myeloperoxidase cells Eo and Neu calculated by myeloperoxidase (MPO) cytochemical reaction in an MPO channel and the fraction of mature PMN measured in the channel for nuclear segmentation of the reflected light beam. It is calculated according to the following formula: DNI% = (Neu%+Eo%) - PMN%. It is measured in percentages.

Table 3 shows the comparative presentation of the mean measured values of WBC and Neu, as well as the mean calculated values of DNI, in the studied men with non-odontogenic abscesses and phlegmons of the neck and in the healthy control subjects.

**Table 3:** Comparison of the studied patients with non-odontogenic abscesses and the control group of healthy men regarding the mean values of WBC, Neu, and DNI WBC: leukocytes; Neu: neutrophils; DNI: delta neutrophil index

	WBC (х10^3^/L)		Neu (х10^3^/L)		DNI (%)	
	Mean value	SD	Mean value	SD	Mean value	SD
Studied group with non-odontogenic abscesses (n=40)	10.19	2.68	7.68	2.76	1.11	0.83
Control group (n=30)	7.37	1.93	4.13	1.48	-1.07	1.22
p-value	p=0.0001		p=0.0001		p=0.0001	

## Discussion

The DNI is the fraction of circulating immature granulocytes and reflects the severity of bacterial infections and septic status in the ill person [13]. It is calculated by subtracting the fraction of mature polymorphonuclear leukocytes from the sum of myeloperoxidase-reactive cells after their numerical value has been determined by a hematological analyzer [14]. In an infection of bacterial etiology, immature neutrophils, such as myelocytes, metamyelocytes, and promyelocytes, but without blasts, enter the peripheral blood after being produced by the bone marrow as a result of enhanced granulocyte production [14,15]. This is the so-called "left-shift" or "left-shifting" phenomenon that underlies the calculation of DNI [15].

The calculated DNI value correlates with the fraction of immature granulocytes in the blood when counted manually and when using an automated hematological cell analyzer when a routine complete blood count is performed [16]. This value is easy to calculate [15,16]. In healthy individuals, the DNI value is 0.0% [13-16]. It can have negative values when the sum of Neu and Eo is less than PMN.

In the patients from our studied group with non-odontogenic abscesses and phlegmons of the neck, the average value of leukocytes is 10.19x10^3^/L, neutrophils is 7.68x10^3^/L, and DNI is 1.11% (Table 3).

In the healthy male control group, the mean measured value of leukocytes is 7.37x10^3^/L, neutrophils is 4.13x10^3^/L, and DNI is -1.07% (Table 3).

The average measured values of WBC and neutrophils in the patients of the studied group are significantly increased compared to the average values of the same parameters in the healthy controls; the same is observed in the measured average values of DNI, and these differences are statistically significant (p<0.05).

The mean plasma concentration of leukocytes in the studied patients is 10.19±2.68x10^3^/L, while the same in healthy men from the control group is 7.37±1.93x10^3^/L, and the difference is statistically significant (p=0.0001).

With regard to the measured average value of neutrophils, it is found that it is significantly higher in patients with non-odontogenic abscesses compared to men in the control group: 7.68±2.76x10^3^/L in the studied group and 4.13±1.48x10^3^/L in the control group (p=0.0001).

Statistically significant (p=0.0001) and almost two times higher is the average calculated value of DNI in the studied men with non-odontogenic abscesses (1.11±0.83%) compared to the control group (-1.07±1.22%).

By themselves, WBC and Neu do not have a definite prognostic informative value in patients with purulent infectious diseases, but after the calculation of DNI and the establishment of significant correlations between DNI and WBC and Neu, the tendency for its prediction in these diseases is marked. The DNI is a calculable marker that is obtained by a specialized method of differentiation of leukocyte fractions by the cytochemical reaction of myeloperoxidase. This method is affected by the concentration of immature granulocytes in the blood. On the other hand, segmented neutrophils, as immature fractions of granulocytes, sharply increase their concentration as a protective response against bacterial irritants and thus participate in the implementation of the inflammatory reaction as a protective mechanism against them. In medical practice, the concentration of immature granulocytes in the peripheral blood correlates with the degree of the inflammatory reaction. It is one of the main indications for determining the severity and prognosis of patients with SIRS [5], with sepsis [6], and with localized inflammatory processes [5-12].

In general, judging by the formula for its calculation, it can be said that DNI is a calculable marker that indicates the degree of inflammation, expressed in the amount of young inflammatory cells in the blood plasma. Therefore, the higher its values, the greater the concentration of these cells, which determine the degree of infection, and the more serious the infectious disease. In this line of thought, DNI can be defined as a predictor of the severity of infection, including in patients with non-odontogenic neck abscesses.

For some of these diseases, DNI reference values (cut-offs) have already been derived. For example, in patients with SIRS and sepsis, they are as follows: DNI between 0.1% and 1.3% is not definitive for the diagnosis; DNI above 1.4% is systemic inflammatory response syndrome; DNI above 2.7% is sepsis; and DNI over 9.3% is severe sepsis [5, 6]; i.e., the higher the DNI value is, the more serious the course and prognosis of the infectious disease are.

The determination of cut-off reference values for DNI in patients with non-odontogenic inflammatory diseases of the neck is in progress, but for now, it is enough to confirm that the marker increases its absolute numerical value in these diseases and can be used in their diagnosis, treatment, and prognosis along with the blood concentration of leukocytes and neutrophils.

The automated process of determining the fractions from which the DNI is then calculated allows the calculation of the marker to be carried out in a short time, which does not waste time for the diagnosis and the determination of the treatment plan in these sometimes life-threatening diseases. At the same time, the cost of performing laboratory tests is significantly reduced because it is calculated from the routinely examined complete blood count of patients without the use of additional consumables [13,16].

## Conclusions

The DNI emerges as a valuable tool for diagnosing, devising treatment strategies, and closely monitoring therapy in non-odontogenic neck abscess patients. Its robust correlation with established inflammation markers, such as leukocyte and neutrophil concentrations, highlights its clinical significance. In addition to its speed and cost-effectiveness, it invites further exploration into specific aspects of DNI and its potential applications within this context. This paves the way for future research to delve deeper into optimizing its utility and addressing any remaining questions within this domain. All this shows that increasing research and analysis in head and neck infectious diseases is yet to come, which is the subject of our future studies in both male and female patients, as well as patients with non-odontogenic and odontogenic abscesses.
